# Common bile duct injury following open conversion of laparoscopic cholecystectomy in 14–15 Weeks pregnancy: A rare case report

**DOI:** 10.1016/j.amsu.2022.104930

**Published:** 2022-11-15

**Authors:** Anita Deborah Anwar, Annisa Dewi Nugrahani, Andi Mulyawan, Dhanny Primantara Johari Santoso, Anita Rachmawati, Muhammad Alamsyah Aziz, Nurhayat Usman, Jusuf Sulaeman Effendi

**Affiliations:** a Department of Obstetrics and Gynaecology, Faculty of Medicine, Universitas Padjadjaran – Dr. Hasan Sadikin General Hospital, Bandung, West Java, Indonesia; b Department of Surgery, Faculty of Medicine, Universitas Padjadjaran – Dr. Hasan Sadikin General Hospital, Bandung, West Java, Indonesia; c Department of Obstetrics and Gynaecology, Slamet General District Hospital, Garut, Faculty of Medicine, Universitas Padjadjaran, West Java, Indonesia

**Keywords:** Case report, CBD injury, Laparoscopic cholecystectomy, Open conversion, Pregnancy, CBD, Common Bile Duct, LapC, Laparoscopic Cholecystectomy, MRCP, Magnetic Resonance Cholangiopancreatography

## Abstract

**Introduction:**

Common bile duct (CBD) injury is the most serious complication of Laparoscopic Cholecystectomy (LapC). Nonetheless, complications of LapC as a treatment for CBD injury are rare in pregnancy. There have been no published case reports regarding complications of CBD injury in gravida patients and their management.

**Case presentation:**

We reported a 32-year-old primiparous woman with CBD injury following open conversion of LapC in 14–15 weeks of pregnancy with enterocutaneous fistula complications. She presented with yellowish fluid leakage from an open wound in her abdomen, and had a history of gallbladder removal and corrective surgery due to bile leakage and intestinal adhesions. Tenderness and serous fluid were found in the area of the previous surgery scar. The laboratory examination showed that the patient was in anaemic condition; Fetal ultrasound examinations showed that the fetus’ condition was within normal limits. The patient was given supportive and medical management with further MRCP plans as well as maternal-fetal close and regular monitoring.

**Clinical discussion:**

In addition to the history and physical examinations, biliary tract imaging holds a pivotal role in this case. LapC is a surgical technique recommended to treat symptomatic cholelithiasis in pregnancies. Despite being rare in pregnancy, prevention of CBD injury by recognizing the pearls and pitfalls of LapC should be done.

**Conclusion:**

Key points for successful treatment of this case are characterized by early recognition of CBD injury, fluid collection and infection control, nutritional balance, and multidisciplinary approaches of the Surgery Department and Obstetrics-Gynaecology Department.

## Highlights

• Complications of laparoscopic cholecystectomy (LapC) are rare in pregnancy.
• Prevention of common bile duct (CBD) injury is a mandatory.
• Multidisciplinary approaches hold an important role for successful treatment.

## 1 Introduction

Pregnancy is a pro-lithogenic state. Gallstones can be detected in 1–3% of pregnant women with the incidence of symptomatic biliary disease during pregnancy ranging from 0.05 to 8%. Moreover, acute cholecystitis is the second most common cause of acute abdomen during pregnancy involving 0.1% cases from all pregnancies [1]. Society of American Gastrointestinal and Endoscopic Surgeons (SAGES) and American College of Obstetrics and Gynaecology (ACOG) recommend the symptomatic pregnant patients for early operative management with Laparoscopic Cholecystectomy (LapC) technique. A recent systematic review study reports that LapC in symptomatic pregnant women has the same level of complications as in symptomatic pregnant patients managed by open cholecystectomy. Thus, LapC is preferred because of its efficacy and efficiency rather than open cholecystectomy [2].

Common bile duct (CBD) injury is the most serious complication of LapC in which the incidence increases when LC becomes the gold standard for treating symptomatic cholelithiasis. Commonly, this event occurs due to anatomical misinterpretation (71–97% of cases). This condition has a significant impact on the patient's quality of life (QoL) and involves medicolegal aspects [3,4]. Early recognition of the occurrence of CBD injury and appropriate management is very important and limiting the delay in diagnosis are essentials for an optimal post-operative outcome, especially in pregnant patients. Open conversion becomes one of the recommended methods to prevent further CBD injury. However, there is a complex complication of CBD injury in a pregnant patient in this case that can lead to giving a significant impact both on the fetus and the mother.

Complications of LapC as a treatment for CBD injury are rare in pregnancy [2]. This case is very rare and is quite challenging because not only CBD injury needs to be considered, but also the patient in this case is in the second trimester of pregnancy, therefore many aspects need to be considered in evaluating the diagnosis, management, and its prognosis. There have been no case reports published regarding CBD Injury complications in gravida patients and how to fix this issue such as reported in this case. Hence, the objective of this study is to report a rare case, to analyse the pearls and pitfalls during LapC in patients with gallstone disease, to evaluate the diagnosis, treatment, and prognosis, especially in pregnant patients so as this study could be used as consideration for further provision and guideline for this case.

## 2 Case presentation

**Fig. 1:**
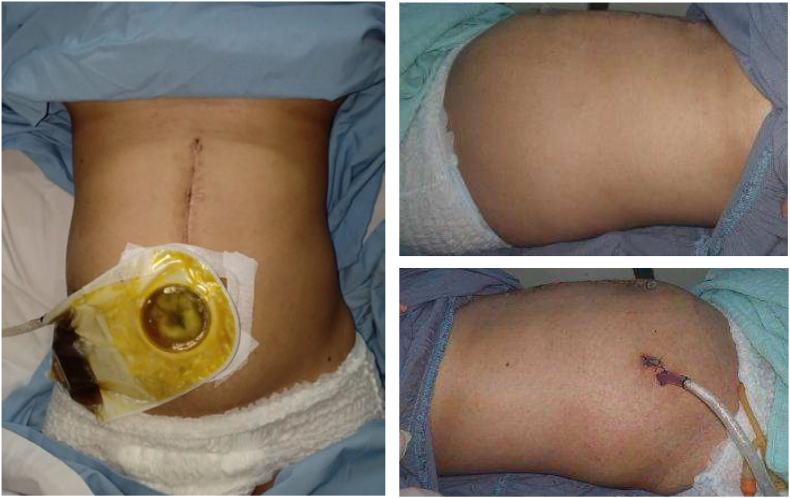
Clinical pictures of the patient. There was an open wound produced a yellowish fluid since about 2 weeks prior to admission in her abdomen.

This following case is described according to the PROCESS guideline [5]. We reported a 32-year-old woman, G2P1A0 (second pregnancy with previous one term parturient history) complained of weakness from two weeks before hospital admission. The complaints had been getting worse since a week prior to admission. Complaints accompanied by intermittent abdominal pain for about 2 weeks and accompanied by a decrease in appetite that had been felt for a month. The patient also complained of an open wound that produced a yellowish fluid for 2 weeks in her abdomen. (Fig. 1). There was a history of nausea and vomiting. No complaints of clay-coloured or tea-coloured defecation were reported. Previously, the patient had a history of gallbladder removal surgery on January 28, 2022, and then, from the patient's abdominal drainage tube came out a yellowish discharge. The patient was treated in the surgery ward at the previous hospital for 12 days. The patient returned home following those treatments and a Magnetic Resonance Cholangiopancreatography (MRCP) examination was performed with the result that there was a localized collection of fluid in the gallbladder. On February 19, 2022, another surgery done was performed again by digestive surgeon in referred hospital due to leakage of bile and intestinal adhesions. This procedure was reported rarely in pregnant woman. Three days after that surgery, the patient complained of yellowish discharge from the surgical wound, and a swab culture was performed with the result of *Pseudomonas* sp. infection. Because the complaints did not improve clinically, the patient went to the emergency ward again for further treatment without took any medication first. No previous personal and family history of gallstone.

From the results of a general examination, the patient was compos mentis with normal vital signs. Tenderness was found in the area of the previous surgery scar on the abdomen. The surgical wound showed an open post-operative wound 4x1x1 centimetres in size, with seroma; no pus and active bleeding. Drainage production was estimated in the range of 20 cc/24 hours (serous).

**Fig. 2:**
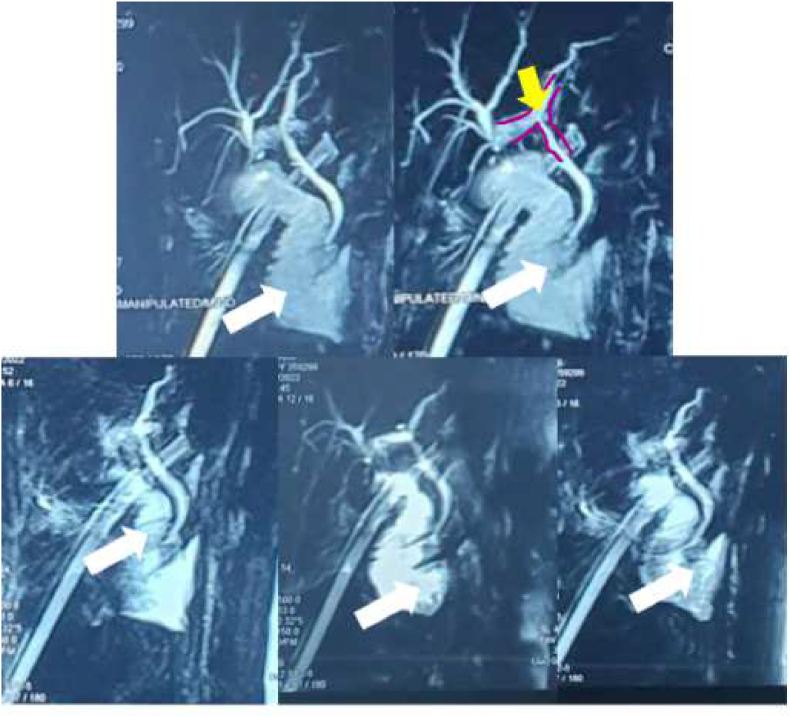
Magnetic Resonance Cholangiopancreatography (MRCP) presents common bile duct (CBD) injury of the patient. White arrow indicates duodenum where yellow arrow indicates area of CBD narrowing (injury) due to laparoscopic cholecystectomy (LapC) procedure. (For interpretation of the references to colour in this figure legend, the reader is referred to the Web version of this article.)

The laboratory examination showed that the patient was in anaemic condition; Haemoglobin level was 7.9 g/dL and a haematocrit level was 24.4%, accompanied by an increase in leukocyte level (13,720/μL). Imaging examination with ultrasound showed the impression of a gallbladder following cholecystectomy and MRCP (Fig. 2) showed that the patient had a CBD narrowing due to LapC procedure. Therefore, the patient was diagnosed with common bile duct injury following open conversion of LapC in 14–15 weeks of pregnancy with enterocutaneous fistula complications with good prognosis for both maternal and fetus.

The patient was given supportive, nutritional, and medical management with Ceftriaxone 1 × 2 grams, Omeprazole 2 × 40 mg, and Ketorolac 3 × 30 mg with adjusted dosage in the following days. Subsequently, consultations were carried out with the Obstetrics and Gynaecology Department regarding the patient's gravida condition. The results from obstetrics and fetal ultrasound examinations showed that the fetus' condition was within normal limits and no anomalies were found. Thus, further close and regular monitoring of maternal condition (drain productions and wound care, as well as further MRCP plans) and fetus condition were also done. No adverse event reported in this patient. Both mother and baby were doing well at the time of this report and accept this management as the best decision for her and her baby. Patient consent was obtained to describe the above findings in the case report in accordance with local legislation.

## 3 Discussion

Pregnancy is a pro-lithogenic state in which estrogen increases cholesterol production, while progesterone reduces the secretion of bile acids and inhibits gall bladder emptying. Gallstones can be detected in 1–3% of pregnant women; The incidence of symptomatic biliary disease during pregnancy ranges from 0.05 to 8%. Acute cholecystitis is the second most common cause of acute stomach during pregnancy with an estimated event of 0.1% [1]. This patient is second-trimester gravida patient, so in addition to the history and physical examination (symptoms and clinical signs), the biliary tract imaging needs to be carried out by: (1) Ultrasound that is non-invasive, does not cause pain in the patient, can be done quickly and safely. Data obtained has a high diagnostic value. There is no contraindication for ultrasound because this examination will not worsen the patient's disease. Ultrasonography is useful for documenting stones in the gallbladder and determining the size of the bile duct; (2) MRCP which provides excellent anatomical details and has a sensitivity and specificity of 95% and 89% respectively, to detect cholelithiasis and choledocholithiasis [[6], [7], [8]].

The next step is the management of these conditions. LapC is a surgical technique recommended by SAGES and ACOG to treat symptomatic cholelithiasis in pregnant patients. LapC is preferred because of its efficacy and efficiency rather than open cholecystectomy [2]. The chosen LapC technique is pneumoperitoneum with the Hasson technique, based on fundal height or the patient's clinical condition. Pregnant women in any trimester can undergo LapC, but it is recommended in the second trimester of pregnancy. Before surgery, a low dose of heparin prophylaxis or low molecular weight heparin (LMWH) can be given to the mother. Maternal monitoring during LapC can be done by assessing the maternal end-tidal volume of PCO_2_ (EtCO_2_) while the fetus is considered to be assessed before and post LapC. Fetal intraoperative monitoring is not recommended [2]. Intraoperative cholangiograms can be done in some patients, usually using low doses, but laparoscopic ultrasonography can be used as an alternative to assess CBD stones. The management of this patient is along with mentioned recommendations.

Common bile duct (CBD) injury is the most serious complication of LapC in which the incidence increases when LC becomes the gold standard for treating symptomatic cholelithiasis. Despite being rare in pregnancy, prevention of CBD injury by recognizing the pearls and pitfalls of LapC should be done [9]. From intra-operative early detection to open conversion such as reported in this case along with close and regular monitoring of maternal and fetus conditions are recommended methods to prevent further maternal-fetal complications.

The patient was approved to undergo surgery for her CBD Injury reconstruction that was followed by conservative treatments related to her enterocutaneous fistula complication and accompanied by close and regular monitoring of the fetus' condition. The patients’ management need multidisciplinary approaches and the collaboration of Surgery and Obstetrics-Gynaecology Department.

## 4 Conclusion

Based on the pearls and pitfalls of this case, any efforts to reduce the risk profile of cholecystectomy in the pregnant patient are appreciated. Key points for successful treatment of this case are characterized by early recognition of CBD injury, control of intra-abdominal fluid collection and infection, nutritional balance, surgical repair by experienced surgeons in biliary reconstruction, and multidisciplinary approaches such as the collaboration of the Surgery Department and Obstetrics-Gynaecology Department.

## Ethical approval

This study does not require an ethical approval as determined by the institutional and departmental review board.

## Source of funding

The study did not receive external funding

## Author contribution

Anita Deborah Anwar (ADA) and Annisa Dewi Nugrahani (ADN) were responsible for the conception and design of the study, interpretation of data, drafting and revising the article to the final form as submitted; She was also involved in the patient's care during hospitalization follow-up. Andi Mulyawan (AM) was responsible for the conception and design of the study, interpretation of data, drafting and revising the article to the final form as submitted; He was also directly involved in the patient's care during hospitalization follow-up. Dhanny Primantara Johari Santoso (DPJS) was involved in conception of design of the study, data interpretation, and editing of the manuscript. Anita Rachmawati (AR) was involved in conception of design of the study, data interpretation, and editing of the manuscript. Muhammad Alamsyah Aziz (MA) was the attending consultant of the patient and contributed directly to patient management as well as conception of design of the study, data interpretation, and editing of the manuscript. Nurhayat Usman (NU) was the attending consultant of the patient and contributed directly to patient management as well as conception of design of the study, data interpretation, and editing of the manuscript. Jusuf Sulaeman Effendi (JSE) was involved in conception of design of the study, data interpretation, and editing of the manuscript.

## Trial registry number

n/a.

## Guarantor

The guarantors of this study is Anita Deborah Anwar, M.D (first author), Annisa Dewi Nugrahani M.D (corresponding author), and Andi Mulyawan, M.D. (second author).

We declare that this manuscript is not under consideration elsewhere and none of the paper's contents have been published previously. All authors have read and approved to the manuscript as written. The authors maintain no conflict of interest.

## Provenance and peer review

Not commissioned, externally peer reviewed.

## Declaration of competing interest

The authors declare that we have no conflicts of interest.
