# Supplementary material for: Common bile duct injury following open conversion of laparoscopic cholecystectomy in 14–15 Weeks pregnancy: A rare case report
Source: Ann Med Surg (Lond). 2022 Nov 15;84:104930. doi: 10.1016/j.amsu.2022.104930 (PMC9793158; doi:10.1016/j.amsu.2022.104930)
Supplement: Multimedia component 1 [file mmc1.docx]

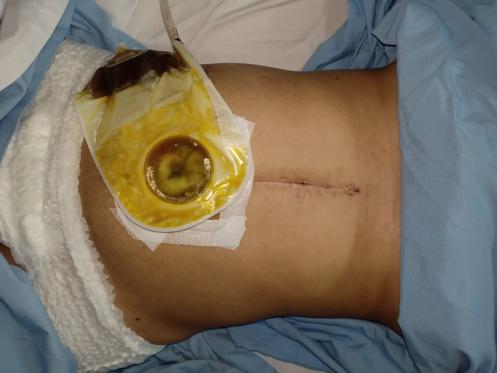

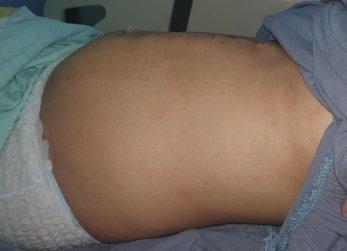

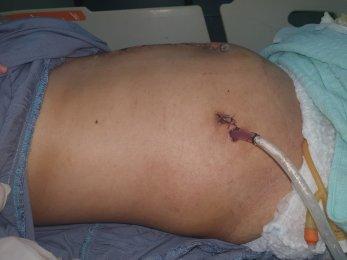


**Figure 1. Clinical pictures of the patient.**

There was an open wound produced a yellowish fluid since about 2 weeks prior to admission in her abdomen.
