# Supplementary material for: Common bile duct injury following open conversion of laparoscopic cholecystectomy in 14–15 Weeks pregnancy: A rare case report
Source: Ann Med Surg (Lond). 2022 Nov 15;84:104930. doi: 10.1016/j.amsu.2022.104930 (PMC9793158; doi:10.1016/j.amsu.2022.104930)
Supplement: Multimedia component 2 [file mmc2.docx]

**
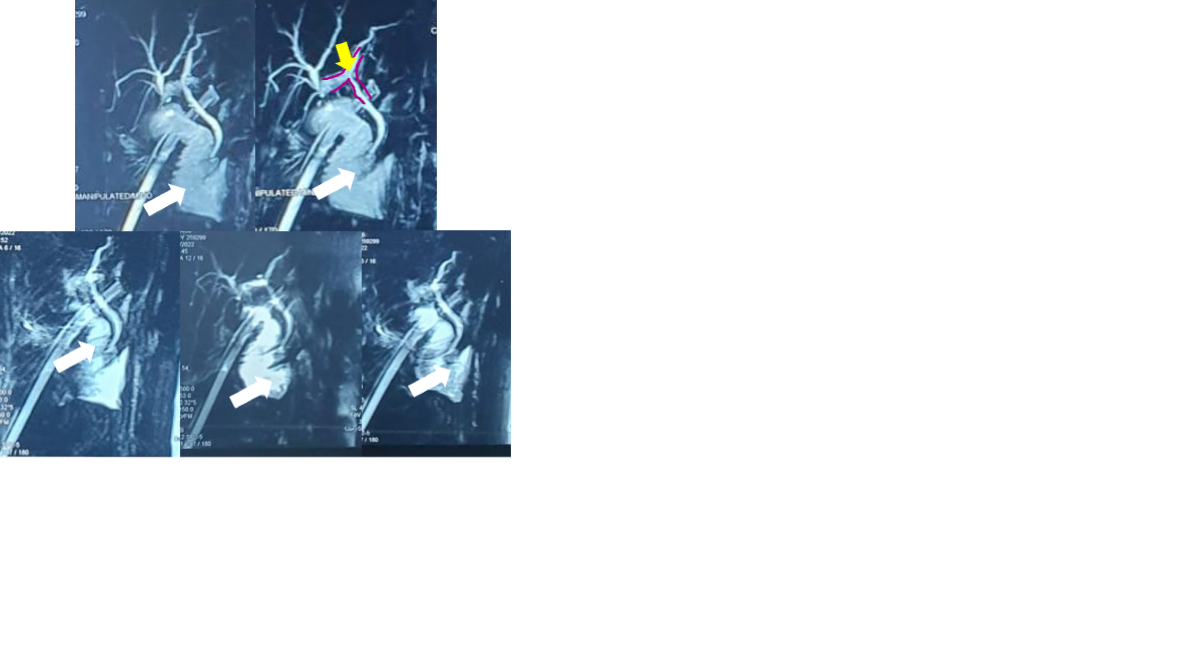
**

**Figure 2. Magnetic Resonance Cholangiopancreatography (MRCP) presents common bile duct (CBD) injury of the patient.**

White arrow indicates duodenum where yellow arrow indicates area of CBD narrowing (injury) due to laparoscopic cholecystectomy (LapC) procedure.
